# Predicting the adoption of evidence-based practice (EBP) based on critical thinking levels among nursing students

**DOI:** 10.1186/s12912-026-04323-6

**Published:** 2026-01-31

**Authors:** Alireza Abdi, Mohammad Mohammadi, Ali Mohammadi, Hosna Ashrafi, Hamzeh Zahabi

**Affiliations:** 1 Department of Emergency and Critical Care Nursing, Faculty of Nursing and Midwifery, Kermanshah University of Medical Sciences, Kermanshah, Iran; 2 Student Research Committee, Faculty of Nursing and Midwifery, Kermanshah University of Medical Sciences, Kermanshah, Iran

**Keywords:** Evidence-based practice adoption, Critical thinking, Nursing students, Nursing

## Abstract

**Aim:**

This study aimed to predict the adoption of EBP based on critical thinking levels among nursing students.

**Background:**

As future healthcare providers, nursing students must acquire essential skills and competencies to transition into clinical practice effectively. Critical thinking and EBP are two influential components that play a pivotal role in this process.

**Methods:**

This descriptive-analytical cross-sectional study was conducted on 300 nursing students. Participants were selected using a convenience sampling method. Data collection tools included a demographic information checklist, an EBP questionnaire, and a critical thinking questionnaire.

**Results:**

A total of 300 students participated in the study, of whom 51.7% were male. The mean age of participants was 22.41 ± 2.01 years, and their average GPA was 16.39 ± 1.17. The mean scores for EBP adoption and critical thinking were 35.48 ± 7.69 (out of 50) and 119.30 ± 23.28 (out of 165), respectively. A strong positive and significant correlation was found between EBP adoption and critical thinking (*r* = 0.677, *p* < 0.001). Linear regression analysis indicated that critical thinking explained 45.6% of the variance in EBP adoption (F = 251.90, *p* < 0.001).

**Discussion:**

The findings underscore that nearly half of the variance in EBP adoption is explained by critical thinking, suggesting the need to prioritize critical thinking development in educational curricula.

**Conclusion:**

The findings suggest that critical thinking can significantly predict EBP adoption among nursing students, explaining 45.6% of the variance. Therefore, it is recommended that nursing education programs emphasize enhancing critical thinking skills to better prepare students for EBP.

## Background

A nurse is a professional equipped with the knowledge and skills required for patient care, treatment, and education. To maintain competence, nurses must continuously update their knowledge by engaging with clinical research [1]. A clinical nurse is considered professional and efficient when they implement care standards in practice, thereby enhancing the quality of care [2]. Evidence-based nursing practice (EBNP) is a process in which nurses use available research evidence, their clinical expertise, and patient specific circumstances to make optimal clinical decisions [3].

The concept of EBP has evolved over several decades. Although it was initially rooted in evidence-based medicine, the field expanded significantly with contributions from nursing scholars [4] .Alan Pearson has made significant contributions to the field of EBP, particularly in nursing and healthcare. He is recognized for his leadership in the development of frameworks and models that facilitate the implementation of EBP in clinical settings [5]. The framework of EBP is anchored on three core components: the best available evidence, clinical expertise, and patient values. Healthcare practitioners utilize these elements to tailor care to individual patient needs while remaining grounded in scientific research [6].

Evidence-based nursing can reduce nursing errors and establish a stable and consistent approach to nursing care [7]. Implementing EBP principles bridges the gap between research and clinical practice, advancing the nursing profession [8]. Importantly, adopting EBP is a prerequisite for its implementation and should be emphasized during nursing education [9]. The American Association of Colleges of Nursing states that EBP is a hallmark of a competent graduate nurse [10]. EBP adoption among nursing students has significant benefits, including improved clinical decision-making, enhanced quality of nursing care, and reduced gaps between education and practice [11].

Clinical challenges encourage active student engagement and promote critical thinking [12]. Critical thinking is a skill that can be enhanced through educational methods such as simulation, problem-based learning (PBL), and group work [13]. As nursing often involves unpredictable situations and diverse patient needs, nurses must move beyond traditional practices to make effective decisions [14]. Thus, an efficient and confident nurse requires skills such as problem-solving and clinical decision-making [15]. One of the primary goals of nursing education is to foster critical thinking among students [16].

critical thinking in nursing is defined as logical and reasoned thinking about problems that may have multiple solutions. It enables nurses to make appropriate decisions in situations where their beliefs and actions diverge [17]. Therefore, the status of critical thinking skills should be regularly assessed in nursing education programs. Critical thinking, as an integral component of education, involves analyzing, evaluating, selecting, and applying knowledge to arrive at the best solutions and this is precisely what a clinical nurse requires [18].

Critical thinking faces obstacles such as classroom environments, teaching methods, and student-teacher ratio [19]. Traditional teaching methods often produce graduates with extensive theoretical knowledge but insufficient ability to address challenging real world issues [20]. Such methods transmit a mix of information and concepts but fail to equip students with the analytical, prioritization, and organizational skills necessary for critical thinking [21]. In contrast, critical thinking can transform academic knowledge into practical skills, bridging the gap between theory and practice [22].

The nursing education system must foster and enhance critical thinking skills among students [23]. Nurses, given the scope and responsibilities of their practice, and the necessity for precise, sensitive, and vital decision-making in providing nursing services, are required not only to acquire knowledge and skills but also to demonstrate sound judgment in critical clinical situations [24].

Despite the recognized importance of critical thinking, numerous studies highlight the weak critical thinking abilities among nursing students in Iran. In a study by Farzi et al. (2023), participants’ critical thinking skills were reported to be at a low level [25]. Research by Gonzalez et al. (2020) and Poodineh Moghaddam et al. (2015) established a correlation between EBP and critical thinking [26, 27]. However, no studies were found specifically examining the relationship between EBP adoption and critical thinking.

Considering that nursing care requires unique solutions tailored to patients, and this necessitates the development of critical thinking skills and EBP in nursing education, this study was conducted to predict the adoption of EBP based on critical thinking levels among nursing students. The hypothesis of this study is that critical thinking skills among students can significantly predict the adoption of EBP.

## Methods

### Study design

This research was a descriptive-analytical cross-sectional study conducted in 2024 among nursing students in Kermanshah, Iran based on the STROBE checklist (supplementary file).

### Participants

The study population included undergraduate nursing students from the Faculty of Nursing and Midwifery at Kermanshah University of Medical Sciences. A sample size of 300 participants was determined based on the Cochran formula and the total population size of 800 students. Participants were selected through convenience sampling. Inclusion criteria included being an undergraduate nursing student, willingness to participate in the study after being informed of its objectives, and completing the questionnaire. Exclusion criteria included failure to answer any of the questionnaire items.

### Study instruments

The study employed three instruments:

1. **Demographic and Academic Information Form**: This included variables such as age, gender, place of residence, GPA, and academic semester.
2. **Standard Evidence-Based Practice (EBP) Adoption Questionnaire**: Developed by Rubin and Parrish (2010), this 10 item questionnaire assesses EBP adoption using a 5 point Likert scale ranging from 1 (“strongly disagree”) to 5 (“strongly agree”). Total scores range from 10 to 50. Its validity was confirmed in a study by Ashktorab et al. through the review of 14 experts, and its reliability was reported with a Cronbach’s alpha of 0.74 [28, 29].
3. **Critical Thinking Skills Questionnaire**: Designed by Ricketts (2003), this 33 item questionnaire measures critical thinking across three components: creativity, maturity, and commitment. Responses are rated on a 5 point Likert scale from 1 (“strongly disagree”) to 5 (“strongly agree”), with total scores ranging from 33 to 165. Component-specific reliability scores were 0.79 for creativity, 0.75 for maturity, and 0.89 for commitment, with an overall reliability score of 0.86. Cultural adaptation and validation of this questionnaire in Iran were conducted by Karami et al. (2014) [30, 31].

### Data collection

With the approval of the study protocol and an introductory letter obtained from the Deputy of Research and Technology of the university, the researchers proceeded to the School of Nursing and Midwifery. Upon presenting the introductory letter to the faculty authorities, the researchers, who were themselves students at the school, received permission to collect data. The researchers then approached the participants in the courtyard and in the classroom and explained the study’s objectives, ensuring the privacy and confidentiality of the participants. Participants were also given the option to complete an online version of the questionnaire via a provided link (https://survey.porsline.ir/s/GqDmt8NZ*).* Assurance was provided that all personal information would remain confidential.

### Data analysis

Descriptive statistics (mean, standard deviation, frequency, and percentage) and inferential statistics (correlation, independent t-test, ANOVA, and linear regression) were used for data analysis. SPSS version 24 was employed for statistical computations. A p-value of less than 0.05 was considered statistically significant for all analyses.

### Ethical considerations

Informed verbal consent was obtained from all participants. Ethical approval for the study was secured from the Research and Technology Department of Kermanshah University of Medical Sciences under the code IR.KUMS.REC.1403.091.

## Results

The subjects were 304 students, from whom 75 filled out the questionnaires through online method. Of the 229 people who filled out the questionnaire in person, 4 people completed the questionnaire incompletely and were excluded from the study. The response rate was 98.6%. The mean age of participants was 22.41 ± 2.01 years, and the average GPA was 16.39 ± 1.17. The gender distribution was nearly equal, with 51.7% male and 48.3% female. Most participants (53%) lived with their families. Seventh-semester students comprised the largest group (21%). The mean scores for EBP adoption and critical thinking were 35.48 ± 7.69 (out of 50) and 119.30 ± 23.28 (out of 165), respectively. The mean scores for the components of critical thinking were 41.40 ± 10.02 for creativity, 31.02 ± 5.89 for maturity, and 46.87 ± 8.87 for commitment (Table 1).

**Table 1:** Demographic and educational characteristics of the subjects

Variable		Number	Percent
Sex	Male	155	51.7
	Female	145	48.3
Residence status	Rented house	7	2.3
	dormitory	134	44.7
	with family	159	53
Semester	1	5	1.7
	2	30	10
	3	30	10
	4	46	15.3
	5	47	15.7
	6	48	16
	7	63	21
	8	31	10.3
Variable		Mean	SD
Age		22.41	2.01
Total average		16.39	1.17

The mean and standard deviation for EBP adoption and critical thinking were analyzed based on gender, but independent t-tests showed no statistically significant differences (*p* > 0.05). Similarly, no significant differences were observed for EBP adoption and critical thinking scores based on participants’ living arrangements, as assessed by ANOVA (*p* > 0.05). The mean and standard deviation for EBP adoption and critical thinking were higher among first and second semester students compared to those in later semesters. According to the ANOVA test, this difference was statistically significant for EBP adoption (*p* = 0.005), but no significant difference was observed for critical thinking (*p* > 0.05) (Table 2).

**Table 2:** The relationship between EBP and CT with demographic and educational variables

Variable		Total EBP (Mean ± SD)	Statistical test	Total CT(Mean ± SD)	Statistical test
Sex	Male	35.34 ± 8.14	t=-0.306 *P* = 0.760	119.40 ± 22.87	t = 0.079 *P* = 0.937
	female	35.62 ± 7.20		119.19 ± 23.79	
Semester	1	40.80 ± 2.16	t = 3.006 *P = 0.005	138.00 ± 7.48	t = 0.839 *P* = 0.556
	2	40.06 ± 7.38		123.10 ± 20.20	
	3	36.46 ± 8.75		119.33 ± 25.43	
	4	34.10 ± 8.49		119.78 ± 25.90	
	5	33.23 ± 7.34		116.17 ± 17.62	
	6	34.43 ± 7.61		118.313 ± 0.44	
	7	35.90 ± 6.16		117.33 ± 19.77	
	8	35.41 ± 7.89		122.16 ± 22.95	
Residence status	Rented house	37.14 ± 5.92	t = 2.179 *P* = 0.115	104.85 ± 33.49	t = 1.603 *P* = 0.203
	dormitory	34.46 ± 8.36		118.67 ± 25.28	
	with family	36.26 ± 7.07		120.47 ± 20.84	

Correlation analysis revealed no significant relationship between age and EBP adoption scores (*r*=-0.017, *p* = 0.774) or critical thinking scores (*r* = 0.003, *p* = 0.995). However, GPA was positively and significantly correlated with both EBP adoption (*r* = 0.272, *p* < 0.001) and critical thinking (*r* = 0.244, *p* < 0.001). A strong, positive, and significant correlation was found between EBP adoption and critical thinking scores (*r* = 0.677, *p* < 0.001) (Table 3).

**Table 3:** Correlation between quantitative variables

Commitment	Growing up	Creativity	Total CT	Total EBP	Total average	Age	Variables	
*r*=-0.014 *p* = 0.811	*r* = 0.033 *p* = 0.574	*r* = 0.001 *p* = 0.991	*r* = 0.003 *p* = 0.955	*r*=-0.017 *p* = 0.774	*r* = 0.129 *p = 0.027		*r* = 1	A
*r* = 0.187 *p < 0.001	*r* = 0.233 *p < 0.001	*r* = 0.264 *p < 0.001	*r* = 0.244 *p < 0.001	*r* = 0.272 *p < 0.001	*r* = 1	*r* = 0.129 *p = 0.027	Total average	
*r* = 0.614 *p < 0.001	*r* = 0.605 *p < 0.001	*r* = 0.673 *p < 0.001	*r* = 0.677 *p < 0.001	*r* = 1	*r* = 0.272 *p < 0.001	*r*=-0.017 *p* = 0.774	Total EBP	
*r* = 0.937 *p < 0.001	*r* = 0.907 *p < 0.001	*r* = 0.961 *p < 0.001	*r* = 1	*r* = 0.677 *p < 0.001	*r* = 0.244 *p < 0.001	*r* = 0.003 *p* = 0.955	Total CT	
*r* = 0.837 *p < 0.001	*r* = 0.836 *p < 0.001	*r* = 1	*r* = 0.961 *p < 0.001	*r* = 0.673 *p < 0.001	*r* = 0.264 *p < 0.001	*r* = 0.001 *p* = 0.991	Creativity	
*r* = 0.772 *p < 0.001	*r* = 1	*r* = 0.836 *p < 0.001	*r* = 0.907 *p < 0.001	*r* = 0.605 *p < 0.001	*r* = 0.233 *p < 0.001	*r* = 0.033 *p* = 0.574	Growing up	
*r* = 1	*r* = 0.772 *p < 0.001	*r* = 0.837 *p < 0.001	*r* = 0.937 *p < 0.001	*r* = 0.614 *p < 0.001	*r* = 0.187 *p < 0.001	*r*=-0.014 *p = 0.811	commitment	

Linear regression analysis indicated that critical thinking accounted for 45.6% of the variance in EBP adoption (F = 251.90, *p* < 0.001) (Table 4).

**Table 4:** Predicting EBP by CT

P value	T value	Coefficient Standard error	B value	Model
< 0.001	15.871	0.014	0.224	Total CT
< 0.001	5.146	1.712	8.809	Constant

Adjusted R^2^ = 45.6 ,F = 251.90, p < 0.001

## Discussion

This study aimed to predict the adoption of EBP based on critical thinking levels among nursing students. The findings revealed that the mean score for EBP adoption among nursing students was 35.48 out of 50, indicating a high level of EBP adoption. Similarly, a study by Abu Bakr et al. (2021) in Jordan found that while most nursing students believed in the importance of EBP, the mean score for its implementation was significantly low [32]. In studies by GEÇİM et al. (2024) in Turkey and Eslami et al. (2024) in Iran, the mean overall score for EBP competence among nursing students was above average [33, 34]. The variation in EBP adoption across countries can be attributed to factors such as access to resources and data, government support, organizational and educational culture, and advancements in technology and innovation [35, 36]. Furthermore, nursing faculty and mentors play a critical role in fostering EBP adoption by presenting clinical questions, sharing the latest research findings, and encouraging research opportunities. These approaches likely enhance students’ engagement with EBP, a factor that appears to be influential at the institution where this study was conducted.

Regarding critical thinking skills, students obtained a mean score of 119.30 out of 165, indicating a high level of critical thinking. Similarly, research by Boso et al. (2021) in Ghana reported that the majority of nursing students had a positive attitude toward critical thinking [37]. In studies conducted by Nemati Vakil Abad et al. (2023) and Fereidouni et al. (2024) in Iran, the mean critical thinking scores of nursing students were reported as moderate and high, respectively [38, 39]. Critical thinking skills are influenced by several factors, including the educational system, a culture of inquiry, and access to modern, high-quality educational resources [40, 41]. According to the researchers of this study, clinical training has a substantial impact on the development of nursing students’ critical thinking skills. In clinical environments, students frequently encounter unpredictable situations, which challenge them to develop the judgment required for effective decision-making in complex clinical contexts.

Analysis by academic semester revealed that first- and second-year students demonstrated higher EBP adoption scores. In contrast, a study by Gonzalez et al. (2021) in Colombia found that ninth-semester students had higher EBP competence scores than tenth-semester students [42]. Similarly, studies by Nantsupawat et al. (2023) in Thailand and Rizalar et al. (2024) in Turkey reported no significant differences in EBP performance scores based on academic year [43, 44]. Higher-semester students are typically exposed to more complex and specialized concepts, requiring the use of scientific evidence to solve problems and improve performance. As they progress through their studies, students tend to develop greater confidence in using scientific evidence, which helps them apply this evidence more effectively in practice [45]. The discrepancy between our findings and those of other studies may be due to the specific conditions at the study site. For instance, higher semester students are often occupied with intensive clinical rotations and practical training, gaining experience, knowledge, and skills but having less time to explore scientific resources. In contrast, lower-semester students may show greater enthusiasm for utilizing academic and research resources, an interest that appears to decline in later semesters. Further research is needed to explore the reasons behind this trend and its implications for EBP adoption among nursing students.

Higher-grade point averages (GPAs) were associated with greater EBP adoption and stronger critical thinking skills. Similar findings were reported in studies by Eslami et al. (2024) in Iran and Nguyen et al. (2023) in Vietnam, indicating a significant positive correlation between GPA and nursing students’ EBP performance and critical thinking skills [34, 46]. However, studies conducted by Al-Ruwaili et al. (2024) in Saudi Arabia, Fereidouni et al. (2024) in Iran, and Shirazi and Heydari (2019) in Iran did not find a significant relationship between GPA and EBP performance or critical thinking [38, 47, 48]. Based on the available evidence, it cannot be definitively concluded that students with higher GPAs necessarily exhibit superior EBP performance or critical thinking abilities. Nevertheless, it appears that students with higher GPAs generally possess stronger analytical skills, which may help them better comprehend and evaluate scientific evidence. Furthermore, such students often exhibit greater motivation and commitment to learning and applying new knowledge. This motivation may encourage them to actively seek out scientific evidence and incorporate it into their practice. Overall, students with higher GPAs tend to have enhanced information analysis capabilities, enabling them to better understand and critique scientific evidence. Additionally, their higher levels of motivation and dedication to acquiring and applying knowledge may drive them to engage more deeply with evidence-based resources in their academic and professional work.

No significant associations were found between age and EBP adoption or critical thinking, a finding consistent with studies by Al-Ruwaili et al. (2024) in Saudi Arabia, Eslami et al. (2024) in Iran, Ness et al. (2022) in Brazil, and Barry et al. (2020) in Iran [34, 47, 49, 50]. However, contrasting results were reported in studies by Nemati Vakilabad et al. (2023) in Iran, Nguyen et al. (2023) in Vietnam, and Sultana and Begum Gul (2021) in Pakistan, where an increase in age was associated with higher scores in EBP performance and critical thinking [39, 46, 51]. As individuals age, they typically accumulate more experience and knowledge, which can contribute to the development of critical thinking skills. Greater life experience enables them to analyze information more effectively and make more logical decisions [52]. However, the varied findings reported across studies suggest that age may have different effects on critical thinking depending on contextual factors. In the present study, older students appeared to have less time to engage with scientific resources due to mental preoccupation with concerns about their future careers and personal life responsibilities, which may explain the lack of a significant relationship between age and critical thinking or EBP performance in this population.

Similarly, gender did not significantly influence EBP adoption or critical thinking. This finding aligns with studies conducted by González et al. (2021) in Colombia, Nantsupawat et al. (2023) in Thailand, and Nguyen et al. (2023) in Vietnam [42, 43, 46]. However, contrasting results were reported in a study by Rizalar et al. (2024) in Turkey, where women scored significantly higher on EBP performance than men [44]. Similarly, Barry et al. (2020) in Iran found a significant relationship between gender and critical thinking, with men scoring higher than women [49]. Based on the reported findings, female students tend to exhibit greater curiosity, attention to detail, responsibility, and engagement with theoretical and professional learning compared to male students [53]. On the other hand, it has been suggested that due to the rational and analytical nature of critical thinking, men may demonstrate higher capabilities in this area. Nevertheless, the importance of emotional regulation in critical thinking suggests that women could surpass men in this domain when emotional control is effectively applied [54]. In the current study, given that critical thinking in nursing is predominantly focused on clinical issues, it appears that gender is not a significant factor influencing critical thinking or EBP performance in this specific context.

A strong, positive correlation was observed between EBP adoption and critical thinking skills (*r* = 0.677), with approximately 45% of the variance in EBP acceptance explained by critical thinking abilities. This finding suggests that students with higher critical thinking skills exhibited greater acceptance of EBP. Similar results were observed in studies by Park and Choi (2024) in South Korea, Melikoglu et al. (2024) in Turkey, and Kim et al. (2024) in South Korea, where 50%,46%, and 20% of the variance in EBP performance, respectively, was predicted by critical thinking skills, supporting the findings of our study [54–56]. Critical thinking fosters curiosity and interest in students, motivating them to seek new knowledge [57]. It enables individuals to examine issues more deeply and comprehensively, pursue innovative and creative solutions, and rely on credible evidence. This process encourages individuals to explore new sciences and studies, leading to a better understanding of the world and the resolution of complex problems [58, 59]. Strategies such as simulation, problem-based learning (PBL), and teamwork have been shown to effectively enhance critical thinking skills in students [13]. Given that critical thinking is an improbable skill, it is recommended that these approaches be integrated into nursing education.

### Limitations

Our study had some limitations, specifically the sampling method was convenience and may affect the generalizability of the study to broader populations.

## Conclusion

This study aimed to predict the acceptance of EBP based on the level of critical thinking among nursing students. The findings revealed a strong, positive correlation between EBP acceptance and critical thinking, with approximately 45% of the variance in EBP acceptance explained by critical thinking skills. Additionally, students with higher grade point averages (GPAs) exhibited greater critical thinking abilities and a higher acceptance of EBP. Given the results of this study and the critical importance of incorporating EBP, it is recommended that educational institutions adopt approaches that strengthen critical thinking skills, which, in turn, enhance EBP acceptance. These strategies can ultimately have a positive impact on patient care in clinical settings, although further research is needed to explore this relationship in greater depth.

### Implication for practice

The results highlight that enhancing critical thinking skills in nursing students can substantially improve their adoption of evidence-based practice, which may lead to better patient care and clinical decision-making. Additionally, we have suggested that future studies explore targeted educational interventions and longitudinal approaches to further examine the causal relationship between critical thinking development and EBP adoption.

## Data Availability

The data that support the findings of this study are available from the corresponding author upon reasonable request.

## Declarations

### AI-assisted

In the preparation of this article, I utilized ChatGPT, an AI language model, to assist with editing and refining the text. The AI contributed to enhancing the clarity, grammar, and overall flow of the manuscript. However, it is important to note that ChatGPT does not contribute to the original ideas or research presented in the article.
